# Theodora (Theo) Colborn: *1927–2014*

**DOI:** 10.1289/ehp.1509743

**Published:** 2015-03-01

**Authors:** Elizabeth Grossman, Laura N. Vandenberg, Kristina Thayer, Linda S. Birnbaum

Theodora Colborn, research scientist and environmental activist, died 14 December 2014 at the age of 87. As the scientist who coined the term “endocrine disruptor,” Theo—as we had the privilege to call her—played a watershed role in the field of environmental health science, particularly in raising public awareness of the effects of chemical contaminants on human health and the environment. It is no exaggeration to say that endocrine disruption science brought about a sea change in how we consider chemical safety and that Theo was instrumental in bringing about this paradigm shift.

The path to environmental health science was neither typical nor direct for Theo. After an early career as a pharmacist, she earned her PhD from the University of Wisconsin at age 58—and never stopped working in science. In the 1980s her research examined the effects of chemical pollutants on the health of Great Lakes wildlife. While synthesizing huge data sets from numerous scientific fields, she found that many Great Lakes species were suffering from health effects that included reproductive and immune system problems and behavioral, hormonal, and metabolic changes. Although the term “endocrine disruptor” was not yet in use, Theo’s descriptions mirrored what we now recognize as characteristics of endocrine disruptor exposures.

Theo’s work helped bring us to our current understanding of the links between the environment and human health. It is now well recognized that environmental exposures to chemical contaminants, even at very low levels, can play a profound role in human health. It is also now understood that the environment plays an important role in most complex diseases. The ability of environmental chemicals to interfere with hormones—particularly the hormones critical to maintaining many vital body systems—may be responsible for many of these health effects. Today’s debates about chemicals such as bisphenol A, glyphosate, and phthalates, as well as ongoing discussions about the origins of diabetes, early-onset puberty, cancers, and neurological disorders, are all part of the science of endocrine disruption. Current conversations about these exposures and health effects, and the state of current scientific research, would not be the same without Theo’s work.

Theo was prescient in recognizing that very low levels of exposure can have profound and lasting health effects, that timing plays a role in determining health outcomes, that environmental exposures can affect several generations, and that severity of a health effect does not always increase with dose in a linear fashion. With these discoveries, endocrine disruption science upset the age-old assumption that “the dose makes the poison.” In doing so it has posed considerable challenges to how we assess and prevent harmful chemical exposures.

Throughout her career, Theo recognized that her ability to understand what was happening to both wildlife and human populations would be limited if she worked only as an individual research scientist. In 1991, as a fellow of the W. Alton Jones Foundation, she brought together a group of 21 scientists with diverse backgrounds to attend the first of a series of conferences at Racine, Wisconsin, that became known simply as “Wingspread.” The Wingspread Consensus Statement (1991), although simple in its wording, made a powerful contribution to the study of environmental contaminants, and the scientists at this meeting—whom Theo referred to as “experts, skeptics, and gurus”—would become the leaders of the field of endocrine disruption.

While staunchly independent and path-breaking, Theo’s work was characterized by collaboration with scientists and researchers from a wide range of disciplines. In 2012 she coauthored an extensive review of the endocrine disruptor literature with 11 other scientists from fields including genetics, environmental epidemiology, neuroendocrinology, and developmental, cell, cancer, and molecular biology. The authorship of this paper included three generations of scientists, from postdocs to professors to Theo herself—a testament to Theo’s commitment to inclusiveness.

At age 76 Theo founded The Endocrine Disruption Exchange (TEDX), a research organization devoted to understanding how environmental exposures to endocrine disruptors interfere with development and health. Her work with TEDX spanned fields including the developmental origins of health and disease, endocrinology, and toxicology. Most recently, her efforts focused on understanding the health effects of chemicals used in the hydraulic fracturing process used to liberate natural gas and oil.

Among the other remarkable aspects of Theo’s work was her ongoing mentorship and encouragement of new scientists, particularly young women scientists. She shaped so many scientists, in so many positive ways. In a 2013 interview she noted her concern for the next generation of science. For those of us fortunate enough to work with her, she was a cheerleader and advisor; she pushed young scientists to look at “the big picture” and not just the data produced in the field or laboratory. Always available for a phone call or to send words of encouragement by e-mail, Theo provided insights and support that helped others achieve their goals. As her colleagues at TEDX noted in a recent message to their supporters, Theo often ended these conversations and e-mails with one word: Onward! For those of us on the receiving end of this word, we were both challenged and uplifted by her spirit and enthusiasm.

The final chapter of her landmark book, *Our Stolen Future* (1996), coauthored with Dianne Dumanoski and John Peterson “Pete” Myers, described the twentieth century as a time of profound change in the relationship between humans and the Earth. “With that transformation, we have been altering the fundamental systems that support life,” they wrote. Given the high stakes, they concluded that solving this dilemma would require not only ingenuity but also courage and caution in the quest to create safer new chemicals and materials.

In so many ways Theo’s work was about caring for the health of future generations. The news she brought us was not good, but it came with tremendous love for the world in all its messy complexities. Her love for science, passion for public health, and willingness to work with a wide diversity of “experts, skeptics, and gurus” has left a permanent mark on those who knew her and shaped the future for generations of scientists who will never have that opportunity.

